# Rationale for Antioxidant Supplementation in Sarcopenia

**DOI:** 10.1155/2012/316943

**Published:** 2012-01-15

**Authors:** Francesco Cerullo, Giovanni Gambassi, Matteo Cesari

**Affiliations:** ^1^Dipartimento di Scienze Gerontologiche, Geriatriche e Fisiatriche, Università Cattolica del Sacro Cuore, Largo Francesco Vito 1, 00168 Roma, Italy; ^2^Institut du Vieillissement, Université de Toulouse, 31000 Toulouse, France

## Abstract

Sarcopenia is an age-related clinical condition characterized by the progressive loss of motor units and wasting of muscle fibers resulting in decreased muscle function. The molecular mechanisms leading to sarcopenia are not completely identified, but the increased oxidative damage occurring in muscle cells during the course of aging represents one of the most accepted underlying pathways. In fact, skeletal muscle is a highly oxygenated tissue and the generation of reactive oxygen species is particularly enhanced in both contracting and at rest conditions. It has been suggested that oral antioxidant supplementation may contribute at reducing indices of oxidative stress both in animal and human models by reinforcing the natural endogenous defenses. Aim of the present paper is to discuss present evidence related to possible benefits of oral antioxidants in the prevention and treatment of sarcopenia.

## 1. Introduction

There is a common and diffuse false myth that aging is synonym of deterioration, pathology, and death. The increased life expectancy in developed and developing countries is parallel to the need to identify interventions able to preserve health and function even at older age, delaying the physical and cognitive declines. Aging is an extremely complex multifactorial process characterized by progressive physiological, genetic, and molecular changes, responsible for the increase risk of morbidity and death [1]. Several hypotheses [2, 3] have been proposed to explain this inborn process common to all living beings, but one of the most plausible and better-accepted currently is the so-called “free radical theory of aging.”

The age-associated loss of skeletal muscle mass and strength (i.e., sarcopenia) seems an unavoidable part of the aging process. After about the age of 50 years, there is a progressive decrease of muscle mass at the rate of 1-2% per year. Similarly but with different decline rate and timing, muscle strength also decreases by about 3% yearly after 60 years of age [4]. Sarcopenia is a multidimensional phenomenon of aging (someone indicates it as a syndrome) and represents a powerful risk factor for the development of negative health-related events in the elderly. In fact, the relationships of sarcopenia with impaired physical performance, frailty, loss of functional independence, and increased risk of falls are all well established in the literature [5]. Moreover, decreased muscle strength is also highly predictive of incident disability and all-cause mortality in older persons [6].

Oxidative damage has been proposed as one of the major contributors of the skeletal muscle decline occurring with aging [7, 8]. The identification of free radicals as promoters of the aging process may imply that their inhibition might limit the detrimental modifications they exert on our organism (and, in particular, on skeletal muscle). In other words, if molecules with antioxidant capacities can counteract the oxidative damage, they may also play a key role in preventing the onset of age-related conditions, including the disabling process [9]. It will come to be true that oxidative damage is at the basis of the pathophysiological mechanisms responsible for sarcopenia (and other geriatric conditions), and interventions aimed at enhancing the endogenous antioxidant defenses (e.g., dietary antioxidant supplementation) may gain special interest.

The purpose of the present paper is to discuss current available evidence about the effects of antioxidant supplementation on sarcopenia. Special attention will be obviously given to studies focused on models of aging and involving older participants.

## 2. The Free Radical Theory of Aging

This theory was formulated for the first time by Harman in 1956 [10]. He proposed that aging and the associated degenerative diseases were consequences of free radical-induced damages to cells and the inability of counterbalancing these changes by endogenous antioxidant defenses. Harman initially explained the production of free radicals through reactions involving molecular oxygen catalyzed in cells by oxidative enzymes and subsequently postulated that genetic and environmental factors might modify this process. In 1972, he then revised his theory identifying the mitochondria as primarily responsible for the physiological process of aging [11]. Since oxidative damage is higher in cells and structures with higher consumption of oxygen, he suggested that mitochondria (consuming most of the intracellular oxygen) were particularly exposed to oxidative damage and potentially affected lifespan. Miquel and colleagues [12] subsequently confirmed such theory by recognizing mitochondria as major actors of cellular aging. More recently and consistently with these concepts, the free radical theory of aging has been switched into a “mitochondrial free radical theory of aging” [13].

Free radicals are a highly reactive chemical species with a single unpaired electron in its outer orbit seeking to pair with another free electron [14]. In particular, reactive oxygen species (ROS), deriving from oxidative metabolism, have higher reactivity than O_2_. ROS are constantly generated in cells of aerobic organisms by the addition of a single electron to the oxygen molecule with subsequently damage of biological macromolecules (like lipids, proteins, and nucleic acids). The interaction of ROS with normal cellular structures leads to potentially nonreversible modifications, with consequent cellular loss of function and death [3, 15, 16].

There are numerous sites of oxidant generation [17]. Mitochondrial electron transport, peroxisomal fatty acid, cytochrome *P*-450, and phagocytic cells (the “respiratory burst”) represent the most important ones. In particular, the main source of ROS (estimated at approximately 90% of the generated total) is located at the inner mitochondrial membrane where oxidative phosphorylation takes place [18]. Moreover, a variety of exogenous stimuli, such as exposure to infections [19], radiations [20], xenobiotics [21], environmental toxins [22], and ultraviolet light [23], may also increase the ROS production *in vivo*. Interestingly, mitochondria are both producers as well as targets of ROS.

In all organisms living in an aerobic environment, ROS play an important role in the maintenance of body homeostasis [24]. Recent studies [25, 26] have even shown that ROS may function as an additional class of cellular messengers, being involved as physiological regulators of intracellular signaling pathways (e.g., response to growth factor stimulation or generation of the inflammatory response to bacterial defense). Therefore, since free radicals are necessary for correct functioning of the human organism, efficient mechanisms of antioxidant defense had to be developed (especially in cells highly exposed to oxidation processes) with the aim of protecting cellular constituents. Endogenous antioxidants are present under diverse and numerous forms, like enzymes (e.g., superoxide dismutase, catalase, glutathione peroxidase, glutathione reductase), vitamins (e.g., vitamin C, vitamin E), and elements (e.g., selenium, zinc). All these substances are able to neutralize ROS and protect cells from free radical damage. Nevertheless, in a normal scenario, about 1% of ROS is still able to avoid the control of the antioxidant system, causing oxidation of surrounding tissues [27]. Moreover, oxidative damage is obviously enhanced when the ROS production increases and/or the antioxidant status decreases. According to the cellular constituents attacked by the oxidative stress, lipid peroxidation [28], protein oxidation [29], and DNA damage [30] will all promote abnormalities of the cell structures incompatible with proper cell function, leading to its death. Such cellular loss has been finally indicated as being responsible for the age-related degenerative diseases and conditions [31].

In summary, according to the free radical theory of aging, oxidative damage is due to a redox imbalance between ROS and the counteracting antioxidant forces generating a vicious cycle responsible for the progressive augmentation of damage [13]. The equilibrium between ROS production, antioxidant defenses, and the cellular structures determines whether a cell exposed to ROS increase will be destined to survival or death [32].

## 3. The Antioxidant Defense System

Antioxidants are substances able to inhibit the rate of oxidation [33, 34]. Mainly, antioxidant enzymes (e.g., catalase, superoxide dismutase (SOD), glutathione peroxidase, glutathione reductase) work to maintain a state of balance preventing the transformation of ROS and to convert them into more stable molecules (like water and molecular oxygen). SOD exists in two forms: Cu/ZnSOD is present in the cytoplasm of the eukaryotic cells whilst MnSOD is located primarily in the mitochondria. Differently, endogenous nonenzymatic elements with antioxidant properties contribute to the maintenance of homeostasis by primarily acting as cofactors for the antioxidant enzymes. A major source of antioxidants is diet [35]. Among dietary antioxidants, the most important (and also largely available as supplements) are vitamin C, vitamin E, and carotenoids.

Vitamin C acts as an antioxidant by inhibiting oxidation. For example, ascorbic acid concentrations are inversely associated with isoprostanes, a marker of lipid peroxidation [36]. Vitamin C is involved in the regeneration of vitamin E in lipoproteins and membranes. In fact, vitamin C reduces the vitamin E radicals generated in cellular membranes and inhibits the propagation of *α*-tocopheroxyl radical. It is the most important hydrophilic antioxidant [27, 37, 38]. Conversely, vitamin E is the most important lipophilic antioxidant. It has been reported a significantly high correlation of *α*-tocopherol (the most diffuse form of circulating vitamin E) with physical performance and of *γ*-tocopherol with skeletal muscle strength [9]. Nunes and colleagues [39] demonstrated that a vitamin-E-deficient diet may cause an increased caspase-like activation (a proapoptotic stimulus) in animal model. Consistently, vitamin E is inversely correlated with lipid peroxidation and positively correlated with in cytochrome oxidase activity (causing a mitochondrial respiratory chain dysfunction) [40].

The supplementation of different antioxidants will provide different effects on oxidation according to the hydrophobicity of the administered molecule. In fact, fat-soluble antioxidants (e.g., vitamin E) are particularly effective at inhibiting lipoprotein peroxidation, whereas water-soluble antioxidants (e.g., vitamin C) more efficiently protect the aqueous phase. However, these antioxidants do not only act individually, but also cooperatively and sometimes even synergistically. Moreover, their radical-scavenging effects depend on various factors, the site of generation of the oxidant and the localization of the antioxidant [41, 42].

Another important source of antioxidants is assured by dietary carotenoids (including *α*-carotene, *β*-carotene, *β*-cryptoxanthin, lutein, zeaxanthin, and lycopene). Carotenoids are lipid soluble molecules that interact with the lipid membrane bilayer [43]. They scavenge free radicals, quench singlet oxygen, inhibit lipid peroxidation, and modulate redox-sensitive transcription factors (e.g., the nuclear factor kappa-light-chain-enhancer of activated B cells or NF-*κ*B) that are involved in the upregulation of proinflammatory cytokines [44, 45]. Upritchard and colleagues [46] have reported that the supplementation of carotenoids may significantly improve antioxidant status and reduce concentrations of isoprostanes.

Numerous foods like fruits, nuts, vegetables, and spices are rich in antioxidants. Blueberries, cranberries, blackberries, plums, apples, cherries, and prunes are fruits particularly rich in antioxidants. Red and black beans, artichokes, and russet potatoes are the vegetables with highest content of antioxidants. Ground cloves, cinnamon, and oregano contain the greatest amount of antioxidants among spices [47].

Finally, it is worth to be mentioned that the antioxidant system function is highly influenced by age [32, 48]. In fact, with aging, there is a progressive decline in mitochondrial function and increase in oxidative damage. ROS overwhelms the endogenous antioxidant defense system during the aging process, causing harmful modifications of myofiber cellular proteins, lipids, and DNA. Moreover, the antioxidant dietary intake may easily decrease for multiple reasons (e.g., mobility disability leading to inadequate food supply; lack of social support; oral problems leading to repetitive and inadequate diets, etc.). Thus, it is not surprising that poor antioxidant status is commonly found at the foundations of several conditions of older age [49].

## 4. Sarcopenia

In 1989, Rosenberg coined the term “sarcopenia” (from Greek: **σαρξ** or flesh and π*εν*ί*α* or loss) to describe the progressive and involuntary loss of skeletal muscle mass and function occurring with aging [50]. Although it is difficult to provide an exact prevalence of sarcopenia due to the heterogeneity of older persons, the existing multiple operative definitions, and diverse (sometimes contradicting) assessment methodologies, common estimates indicate it as 5–13% in the 60–70-year-old age group and 11–50% in the 80-year and older age group [51]. Such figures are expected to dramatically rise in the next future due to the progressive aging of Western populations with a profound and negative impact on public health. In fact, sarcopenia (one of the most important geriatric syndromes) represents an important risk factor for functional impairment [52], physical disability [53, 54], falls [55], and frailty [56, 57].

Skeletal muscle is a tissue formed by multiple types of fibers. Briefly, type I fibers are slow contracting and use an oxidative metabolism. Differently, type II fibers are fast contracting and mainly glycolytic. Sarcopenic muscle mass reduction is primarily due to a loss of muscle fibers particularly characterized by a preferential atrophy of type II fibers. At the same time, a conversion of fast type II muscle fibers into slow type I fibers (with resulting loss in muscle power and decline in protein synthesis, especially of myosin heavy chains) has been described [58–60]. Overall, these changes lead to a smaller, slower contracting muscle with resulting reduced capacity to adequately perform activities of daily living. These anatomical modifications have been (at least partly) attributed to the age-related increase of oxidative damage. In fact, the skeletal muscle is the largest consumer of oxygen in the body with muscle fibers continuously generating ROS (especially during the contractile activity). Studies adopting muscle biopsies have confirmed that markers of oxidative damage are particularly and locally elevated in skeletal muscle of older adults [61–64], promoting the above-mentioned inadequacy of the antioxidant system in preventing damages [32, 65]. Some components of the enzymatic scavenger system, such as catalase, glutathione transferase, and superoxide dismutase, are also significantly depressed in elderly muscle. The consequent prooxidant status results in the alteration of mitochondrial DNA and abnormalities in the electron transport system, leading to reduced calcium uptake by the sarcoplasmic reticulum [66], irreversible damage of the cell, and its consequent death [67–69].

In the healthy muscle, proteins and amino acids are ideally balanced between synthesis and breakdown. In the elderly, this equilibrium is disrupted because of a lower synthesis and an increased breakdown rate of myofibrillar and mitochondrial proteins. Several endogenous and exogenous factors may affect the organism capacity to maintain the protein homeostasis. For example, it has been hypothesized that muscle decline might be caused by the direct detrimental effects of the chronic low-grade inflammatory status of advanced age. In this context, it cannot be ignored the close relationship between inflammation and oxidative damage. Interestingly, both inflammation (in particular through the TNF-*α* pathway) and oxidative damage are major regulator of cellular apoptosis and protein metabolism. At the same time, the age-related muscle protein loss may also macroscopically be caused by the frequent reduction of food intake in elders. Older persons are particularly exposed to the risk of (micro- and macronutrient) malnutrition that might also be related to endogenous (e.g., malabsorption, edentulism) or exogenous (e.g., lack of social support, disability) causes. Consequently, antioxidant supplementation represents a promising intervention potentially able to correct the inadequate diet of older persons and prevent the skeletal muscle decline by inhibiting the vicious cycle at the basis of protein catabolism.

Antioxidant supplementation may not replace the age-related decline of the more complex antioxidant enzymatic system. Nevertheless, in theory, by reinforcing the antioxidant nonenzymatic defences, supplementation might still be helpful in preventing the onset of age-related conditions (including sarcopenia) by acting on the same cause (i.e., oxidation). However, further studies are needed to better understand the relationship between nonenzymatic and enzymatic antioxidant defenses. In particular, it could be that the two components are not parallel and independent, but indeed synergistically constitute the antioxidant system. Interestingly, Selman and colleagues [70] recently demonstrated that vitamin C supplementation in mice is associated with a downregulation of antioxidant protection genes (including MnSOD).

## 5. Antioxidant Supplementation and Sarcopenia

Despite the clinical relevance of sarcopenia and the large interest for antioxidant supplementation (both from a research and commercial standpoint of view), evidence in this field is extremely limited and controversial. Most of the studies available in the literature are from epidemiological data with most even coming from cross-sectional observations.

The absence of a unique operative definition of sarcopenia represents a major issue limiting the conduction of research on the topic. In fact, most of the human studies analyze the relationship between dietary antioxidant supplementation and physical performance or muscle strength measures, without specifically focusing on the broader condition of sarcopenia. In fact, the quantitative (i.e., muscle mass) and qualitative (i.e., muscle strength, muscle function) declines follow separate and different trajectories with aging. The need of combining them into one single bidimensional definition of sarcopenia is critical from a theoretical and methodological perspective and has to be taken into account when specifically facing the topic sarcopenia. In other words, the separate evaluation of muscle mass or muscle strength may significantly affect the study of sarcopenia (limiting its exploration to only one of the two components) and likely result in biased findings. To our knowledge, there are currently no trials verifying the effects of antioxidant supplementation on sarcopenia (as identified by one of several the consensus definitions provided by international groups of experts). Interestingly, a recent statement from the Society on Sarcopenia, Cachexia, and Wasting Disease does not even mention antioxidant supplementation as a possible tool to manage sarcopenia in older persons [71].

Several studies have been conducted to evaluate the effects of antioxidant supplementation on antioxidant status [72, 73]. Overall, results consistently report significant improvements of antioxidant biomarkers after a period of specific supplementation.

Differently, the effect of antioxidant supplementation on muscle performance is still and largely controversial. Here are just a few examples of positive studies (from both animal and human models) among the large body of literature suggesting a beneficial effect of antioxidant supplementation and sarcopenia. Jakeman and Maxwell [74] showed a protective effect of vitamin C supplementation against exercise-induced muscle damage. Similarly, Shafat and colleagues [75] reported a reduction of muscle damage by adopting a counteracting vitamin E and C supplementation. An antioxidant mixture containing vitamin E, vitamin A, rutin, zinc, selenium has shown to increase the anabolic response of old muscle to leucine and the leucine-induced inhibition of protein degradation in rats [76]. Resveratrol, a natural polyphenol found in grapes, peanuts, and berries [77], has shown a protective effect against oxidative stress in skeletal muscle through the expression of antioxidant enzymes [78]. At the same time, a similar (possibly larger) number of studies have reported negative results on the topic. For example, some trials have reported that antioxidant supplementation may even be unfavorable on physical performance and underlying biological mechanisms [79, 80]. A decrease of baseline levels of antioxidant mitochondrial enzymes was reported by Strobel and colleagues [81] after 14 weeks of vitamin E and *α*-lipoic acid administration in rats. Data by Ristow and colleagues [82] suggested that oral administration of ascorbic acid and *α*-tocopherol prevents exercise training-induced increases in insulin sensitivity and ROS defense capacity. Consistently, the work by Higashida and colleagues [83] showed no inhibitory effect on the adaptive responses of muscle to exercise by antioxidant vitamin supplementation. Several studies [84–86] showed no effects of antioxidants supplementation on muscle function after exercise-induced muscle damage. In a recent work, Theodorou and colleagues [87] found that a supplementation with ascorbic acid and tocopherol does not affect muscle performance. Kondratov and colleagues [88] investigated possible improvements of signs of premature aging (e.g., cataracts, cornea inflammation, joint ossification, and muscle waste) in BMAL1 knockout mice by supplementation of N-acetyl-L-cysteine (NAC), a low-molecular-weight antioxidant. Result suggested an extended lifespan, but excluded significant effects on sarcopenia. Sacheck et al. [89] showed *α*-tocopherol supplementation in older fit men did not suppress postexercise elevations in biomarkers of muscle damage and lipid peroxidation such as in younger men. More recently, vitamin E and C supplementation have shown to reduce muscular levels of oxidative stress in repetitively loaded muscles of old rats, but no increase in muscle mass and maximal force production (after more than 4 weeks of training) was found [90]. Consistent results were obtained by Barker and colleagues [91] after administration of vitamin E and C in men undergoing anterior cruciate ligament surgical repair. It might seem that the biological effects of antioxidant supplementations are easily captured as increased levels of antioxidant biomarkers. Differently, the clinically relevant and beneficial effects of antioxidant supplementation are more difficult to be obtained (always if obtainable!).

Possible reasons for these controversial results are various. First of all, it is possible that sarcopenia is not related to oxidative damage, so that the obtained negative results are indeed “true negative” findings. Second, it is not automatic that the modification of a biomarker concentrations is able to parallely change clinical parameters. It is more likely that subclinical effects are more sensible to changes than clinically evident manifestations. And this is particularly true when testing interventions in an extremely complex field as geriatric syndromes, in which one sign/symptom is not necessarily indicative of a well-defined condition. Different types and doses of antioxidants administered, timing of supplementation, the adopted animal species, and the measured biomarkers may represent other important causes of the negative findings. As mentioned, the heterogeneity and methodological limitations affecting the study of sarcopenia may further explain the controversial findings.

Finally, it is noteworthy the extreme scarcity of available clinical trials in humans on this topic. In fact, most of the positive results are obtained in animal models and still wait to be confirmed in humans. Furthermore, recent studies [92] reporting possible negative effects (e.g., cardiovascular and all-cause mortality) of long-term, high-dosage antioxidant supplementation (in particular, for vitamin E) cannot be ignored.

In the present paper, we chose not to go into many details about posologies of antioxidant supplementations. Current literature on the topic is extremely heterogeneous for methodological approaches, study designs (i.e., *in vivo*, *in vitro*, epidemiological evaluations, clinical trials, animal or human models), interventions (single antioxidant molecules or in combination, timing of administration, posologies), and outcome measures (i.e., biomarkers of muscle decline). Such heterogeneity is not surprising considering the relative novelty of the topic. After all, sarcopenia is a condition theoretically defined only about 20 years ago, but its operative definition is still debated.

## 6. Conclusion

In summary, there is some evidence that oral antioxidant supplementation may reduce muscle damage, but experimental results are largely preliminary and far to be clinically relevant, at least, as suggestive of positive benefits. In fact, a large body of evidence may indicate extreme cautiousness in taking antioxidant supplementation as preventive measures against aging process and age-related conditions. Further studies are needed to support the widespread practice of oral antioxidant supplementation and to determine appropriate recommendations in elderly.

Although antioxidant supplementation through diet is receiving growing attention, supporting evidence is still scarce and equivocal. Antioxidant supplementation could benefit muscle protein metabolism during aging, but further trials in humans and with adequate sample sizes are required to clearly establish the hypothesized relationship between antioxidants and sarcopenia. In this context, a better understanding of oxidation mechanisms, timing and doses of antioxidant supplementation, and appropriate methodological approaches to study this theme is needed to provide convincing evidence and justify the current widespread use of antioxidants supplementation.

## References

[1] Harman D (2003). The free radical theory of aging. *Antioxid Redox Signal*.

[2] Medvedev ZA (1990). An attempt at a rational classification of theories of ageing. *Biological Reviews of The Cambridge Philosophical Society*.

[3] Weinert BT, Timiras PS (2003). Theories of aging. *Journal of Applied Physiology*.

[4] von Haehling S, Morley JE, Anker SD (2010). An overview of sarcopenia: facts and numbers on prevalence and clinical impact. *The Journal of Cachexia, Sarcopenia and Muscle*.

[5] Roubenoff R (2000). Sarcopenia and its implications for the elderly. *European Journal of Clinical Nutrition*.

[6] Metter EJ, Talbot LA, Schrager M, Conwit R (2002). Skeletal muscle strength as a predictor of all-cause mortality in healthy men. *The Journals of Gerontology Series A*.

[7] Pansarasa O, Castagna L, Colombi B, Vecchiet J, Felzani G, Marzatico F (2000). Age and sex differences in human skeletal muscle: role of reactive oxygen species. *Free Radical Research*.

[8] Fanò G, Mecocci P, Vecchiet J (2001). Age and sex influence on oxidative damage and functional status in human skeletal muscle. *Journal of Muscle Research and Cell Motility*.

[9] Cesari M, Pahor M, Bartali B (2004). Antioxidants and physical performance in elderly persons: the Invecchiare in Chianti (InCHIANTI) study. *The American Journal of Clinical Nutrition*.

[10] Harman D (1956). Aging: a theory based on free radical and radiation chemistry. *The Journals of Gerontology*.

[11] Harman D (1972). The biologic clock: the mitochondria?. *The Journal of The American Geriatrics Society*.

[12] Miquel J, Economos AC, Fleming J, Johnson JE (1980). Mitochondrial role in cell aging. *Experimental Gerontology*.

[13] Sastre J, Pallardó FV, García de la Asunción J, Viña J (2000). Mitochondria, oxidative stress and aging. *Free Radical Research*.

[14] Halliwell BH, Gutteridge JMC (1989). *Free Radicals in Biology and Medicine*.

[15] Beckman KB, Ames BN (1998). The free radical theory of aging matures. *Physiological Reviews*.

[16] Rossi P, Marzani B, Giardina S, Negro M, Marzatico F (2008). Human skeletal muscle aging and the oxidative system: cellular events. *Current Aging Science*.

[17] Gil Del Valle L Oxidative stress in aging: theoretical outcomes and clinical evidences in humans.

[18] Balaban RS, Nemoto S, Finkel T (2005). Mitochondria, oxidants, and aging. *Cell*.

[19] Schwarz KB (1996). Oxidative stress during viral infection: a review. *Free Radical Biology & Medicine*.

[20] Riley PA (1994). Free radicals in biology: oxidative stress and the effects of ionizing radiation. *International Journal of Radiation Biology*.

[21] Pagano G (2002). Redox-modulated xenobiotic action and ROS formation: a mirror or a window?. *Human & Experimental Toxicology*.

[22] Shi H, Shi X, Liu K (2004). Oxidative mechanism of arsenic toxicity and carcinogenesis. *Molecular and Cellular Biochemistry*.

[23] Scharffetter-Kochanek K, Wlaschek M, Brenneisen P, Schauen M, Blaudschun R, Wenk J (1997). UV-induced reactive oxygen species in photocarcinogenesis and photoaging. *The Journal of Biological Chemistry*.

[24] Gemma C, Vila J, Bachstetter A, Bickford PC, Riddle DR (2007). Oxidative stress and the aging brain: from theory to prevention. *Brain Aging: Models, Methods, and Mechanisms*.

[25] Knapp LT, Klann E (2002). Role of reactive oxygen species in hippocampal long-term potentiation: contributory or inhibitory?. *Journal of Neuroscience Research*.

[26] Finkel T (2011). Signal transduction by reactive oxygen species. *Journal of Cell Biology*.

[27] Fusco D, Colloca G, Lo Monaco MR, Cesari M (2007). Effects of antioxidant supplementation on the aging process. *Journal of Clinical Interventions in Aging*.

[28] Schafer FQ, Buettner GR (2000). Acidic pH amplifies iron-mediated lipid peroxidation in cells. *Free Radical Biology & Medicine*.

[29] Stadtman ER (2004). Role of oxidant species in aging. *Current Medicinal Chemistry*.

[30] Kil IS, Huh TL, Lee YS, Lee YM, Park JW (2006). Regulation of replicative senescence by NADP^+^-dependent isocitrate dehydrogenase. *Free Radical Biology & Medicine*.

[31] Goswami A, Dikshit P, Mishra A, Mulherkar S, Nukina N, Jana NR (2006). Oxidative stress promotes mutant huntingtin aggregation and mutant huntingtin-dependent cell death by mimicking proteasomal malfunction. *Biochemical and Biophysical Research Communications*.

[32] Kregel KC, Zhang HJ (2007). An integrated view of oxidative stress in aging: basic mechanisms, functional effects, and pathological considerations. *American Journal of Physiology—Regulatory, Integrative and Comparative Physiology*.

[33] Trachootham D, Lu W, Ogasawara MA (2008). Redox regulation of cell survival. *Antioxidants & Redox Signaling*.

[34] Clarkson PM, Thompson S (2000). Antioxidants: what role do they play in physical activity and health?. *American Journal of Clinical Nutrition*.

[35] Berger MM (2005). Can oxidative damage be treated nutritionally?. *Clinical Nutrition*.

[36] Block G, Dietrich M, Norkus EP (2002). Factors associated with oxidative stress in human populations. *American Journal of Epidemiology*.

[37] Niki E, Yamamoto Y, Takahashi M (1988). Free radical-mediated damage of blood and its inhibition by antioxidants. *Journal of Nutritional Science and Vitaminology*.

[38] Frei B, England L, Ames BN (1989). Ascorbate is an outstanding antioxidant in human blood plasma. *Proceedings of the National Academy of Sciences of the United States of America*.

[39] Nunes VA, Gozzo AJ, Juliano MA (2003). Antioxidant dietary deficiency induces caspase activation in chick skeletal muscle cells. *Brazilian Journal of Medical and Biological Research*.

[40] Rafique R, Schapira AH, Coper JM (2004). Mitochondrial respiratory chain dysfunction in ageing; influence of vitamin E deficiency. *Free Radical Research*.

[41] Niki E, Noguchi N, Tsuchihashi H, Gotoh N (1995). Interaction among vitamin C, vitamin E, and beta-carotene. *American Journal of Clinical Nutrition*.

[42] Rinne T, Mutschler E, Wimmer-Greinecker G, Moritz A, Olbrich HG (2000). Vitamins C and E protect isolated cardiomyocytes against oxidative damage. *International Journal of Cardiology*.

[43] Semba D, Lauretani F, Ferrucci L (2007). Carotenoids as protection against sarcopenia in older adults. *Archives of Biochemistry and Biophysics*.

[44] Hu P, Reuben DB, Crimmins EM, Harris TB, Huang MH, Seeman TE (2004). The effects of serum beta-carotene concentration and burden of inflammation on all-cause mortality risk in high-functioning older persons: MacArthur studies of successful aging. *The Journals of Gerontology Series A*.

[45] Walston J, Xue Q, Semba RD (2006). Serum antioxidants, inflammation, and total mortality in older women. *American Journal of Epidemiology*.

[46] Upritchard JE, Schuurman CR, Wiersma A (2003). Spread supplemented with moderate doses of vitamin E and carotenoids reduces lipid peroxidation in healthy, nonsmoking adults. *American Journal of Clinical Nutrition*.

[47] Wu X, Beecher GR, Holden JM, Haytowitz DB, Gebhardt SE, Prior RL (2004). Lipophilic and hydrophilic antioxidant capacities of common foods in the United States. *Journal of Agricultural and Food Chemistry*.

[48] Petersen KF, Befroy D, Dufour S (2003). Mitochondrial dysfunction in the elderly: possible role in insulin resistance. *Science*.

[49] Maxwell SRJ (1995). Prospects for the use of antioxidant therapies. *Drugs*.

[50] Rosenberg IH (1997). Sarcopenia: origins and clinical relevance. *Journal of Nutrition*.

[51] Waters DL, Baumgartner RN, Garry PJ, Vellas B (2010). Advantages of dietary, exercise-related, and therapeutic interventions to prevent and treat sarcopenia in adult patients: an update. *Journal of Clinical Interventions in Aging*.

[52] Janssen I, Heymsfield SB, Ross R (2002). Low relative skeletal muscle mass (sarcopenia) in older persons is associated with functional impairment and physical disability. *Journal of the American Geriatrics Society*.

[53] Baumgartner RN, Koehler KM, Gallagher D (1998). Epidemiology of sarcopenia among the elderly in New Mexico. *American Journal of Epidemiology*.

[54] Janssen I, Shepard DS, Katzmarzyk PT, Roubenoff R (2004). The healthcare costs of sarcopenia in the United States. *Journal of the American Geriatrics Society*.

[55] Wolfson L, Judge J, Whipple R, King M (1995). Strength is a major factor in balance, gait, and the occurrence of falls. *The Journals of Gerontology Series A*.

[56] Vanitallie TB (2003). Frailty in the elderly: contributions of sarcopenia and visceral protein depletion. *Metabolism*.

[57] Bauer JM, Sieber CC (2008). Sarcopenia and frailty: a clinician's controversial point of view. *Experimental Gerontology*.

[58] Akima H, Kano Y, Enomoto Y (2001). Muscle function in 164 men and women aged 20–84 yr. *Medicine & Science in Sports & Exercise*.

[59] Burton LA, Sumukadas D (2010). Optimal management of sarcopenia. *Journal of Clinical Interventions in Aging*.

[60] Morley JE, Baumgartner RN, Roubenoff R, Mayer J, Nair KS (2001). Sarcopenia. *Journal of Laboratory and Clinical Medicine*.

[61] Pansarasa O, Bertorelli L, Vecchiet J, Felzani G, Marzatico F (1999). Age-dependent changes of antioxidant activities and markers of free radical damage in human skeletal muscle. *Free Radical Biology and Medicine*.

[62] Mecocci P, Fanó G, Fulle S (1999). Age-dependent increases in oxidative damage to DNA, lipids, and proteins in human skeletal muscle. *Free Radical Biology & Medicine*.

[63] Lim PS, Cheng YM, Wei YH (2002). Increase in oxidative damage to lipids and proteins in skeletal muscle of uremic patients. *Free Radical Research*.

[64] Gianni P, Jan KJ, Douglas MJ, Stuart PM, Tarnopolsky MA (2004). Oxidative stress and the mitochondrial theory of aging in human skeletal muscle. *Experimental Gerontology*.

[65] Palomero J, Jackson MJ (2010). Redox regulation in skeletal muscle during contractile activity and aging. *Journal of Animal Science*.

[66] Fulle S, Protasi F, Di Tano G (2004). The contribution of reactive oxygen species to sarcopenia and muscle ageing. *Experimental Gerontology*.

[67] Reid MB, Li YP (2001). Cytokines and oxidative signalling in skeletal muscle. *Acta Physiologica Scandinavica*.

[68] Dirks A, Leeuwenburgh C (2002). Apoptosis in skeletal muscle with aging. *American Journal of Physiology—Regulatory, Integrative and Comparative Physiology*.

[69] Kim JS, Wilson JM, Lee SR (2010). Dietary implications on mechanisms of sarcopenia: roles of protein, amino acids and antioxidants. *The Journal of Nutritional Biochemistry*.

[70] Selman C, McLaren JS, Meyer C (2006). Life-long vitamin C supplementation in combination with cold exposure does not affect oxidative damage or lifespan in mice, but decreases expression of antioxidant protection genes. *Mechanisms of Ageing and Development*.

[71] Morley JE, Abbatecola AM, Argiles JM (2011). Sarcopenia with limited mobility: an international consensus. *Journal of the American Medical Directors Association*.

[72] Gupta AD, Dhundasi SA, Ambekar JG, Das KK (2007). Effect of l-ascorbic acid on antioxidant defense system in testes of albino rats exposed to nickel sulfate. *Journal of Basic and Clinical Physiology and Pharmacology*.

[73] Rodrigo R, Prat H, Passalacqua W, Araya J, Bächler JP (2008). Decrease in oxidative stress through supplementation of vitamins C and E is associated with a reduction in blood pressure in patients with essential hypertension. *Clinical Science*.

[74] Jakeman P, Maxwell S (1993). Effect of antioxidant vitamin supplementation on muscle function after eccentric exercise. *European Journal of Applied Physiology and Occupational Physiology*.

[75] Shafat A, Butler P, Jensen RL, Donnelly AE (2004). Effects of dietary supplementation with vitamins C and E on muscle function during and after eccentric contractions in humans. *European Journal of Applied Physiology*.

[76] Marzani B, Balage M, Vénien A (2008). Antioxidant supplementation restores defective leucine stimulation of protein synthesis in skeletal muscle from old rats. *Journal of Nutrition*.

[77] Baur JA, Sinclair DA (2006). Therapeutic potential of resveratrol: the in vivo evidence. *Nature Reviews Drug Discovery*.

[78] Jackson JR, Ryan MJ, Hao Y, Alway SE (2010). Mediation of endogenous antioxidant enzymes and apoptotic signaling by resveratrol following muscle disuse in the gastrocnemius muscles of young and old rats. *American Journal of Physiology—Regulatory, Integrative and Comparative Physiology*.

[79] Marshall RJ, Scott KC, Hill RC (2002). Supplemental vitamin C appears to slow racing greyhounds. *Journal of Nutrition*.

[80] Gomez-Cabrera MC, Domenech E, Romagnoli M (2008). Oral administration of vitamin C decreases muscle mitochondrial biogenesis and hampers training-induced adaptations in endurance performance. *American Journal of Clinical Nutrition*.

[81] Strobel NA, Peake JM, Matsumoto A, Marsh SA, Coombes JS, Wadley GD (2011). Antioxidant supplementation reduces skeletal muscle mitochondrial biogenesis. *Medicine & Science in Sports & Exercise*.

[82] Ristow M, Zarse K, Oberbach A (2009). Antioxidants prevent health-promoting effects of physical exercise in humans. *Proceedings of the National Academy of Sciences of the United States of America*.

[83] Higashida K, Kim SH, Higuchi M, Holloszy JO, Han DH (2011). Normal adaptations to exercise despite protection against oxidative stress. *American Journal of Physiology—Endocrinology and Metabolism*.

[84] Bailey DM, Williams C, Betts JA, Thompson D, Hurst TL (2011). Oxidative stress, inflammation and recovery of muscle function after damaging exercise: effect of 6-week mixed antioxidant supplementation. *European Journal of Applied Physiology*.

[85] Close GL, Ashton T, Cable T (2006). Ascorbic acid supplementation does not attenuate post-exercise muscle soreness following muscle-damaging exercise but may delay the recovery process. *British Journal of Nutrition*.

[86] Petersen EW, Ostrowski K, Ibfelt T (2001). Effect of vitamin supplementation on cytokine response and on muscle damage after strenuous exercise. *American Journal of Physiology—Cell Physiology*.

[87] Theodorou AA, Nikolaidis MG, Paschalis V (2011). No effect of antioxidant supplementation on muscle performance and blood redox status adaptations to eccentric training. *The American Journal of Clinical Nutrition*.

[88] Kondratov RV, Vykhovanets O, Kondratova AA, Antoch MP (2009). Antioxidant N-acetyl-L-cysteine ameliorates symptoms of premature aging associated with the deficiency of the circadian protein BMAL1. *Aging*.

[89] Sacheck JM, Milbury PE, Cannon JG, Roubenoff R, Blumberg JB (2003). Effect of vitamin E and eccentric exercise on selected biomarkers of oxidative stress in young and elderly men. *Free Radical Biology and Medicine*.

[90] Ryan MJ, Dudash HJ, Docherty M (2010). Vitamin E and C supplementation reduces oxidative stress, improves antioxidant enzymes and positive muscle work in chronically loaded muscles of aged rats. *Experimental Gerontology*.

[91] Barker T, Leonard SW, Hansen J (2009). Vitamin E and C supplementation does not ameliorate muscle dysfunction after anterior cruciate ligament surgery. *Free Radical Biology and Medicine*.

[92] Gee PT (2011). Unleashing the untold and misunderstood observations on vitamin E. *Genes & Nutrition*.

